# Hybrid representation learning for human m^6^A modifications with chromosome-level generalizability

**DOI:** 10.1093/bioadv/vbaf170

**Published:** 2025-07-14

**Authors:** Muhammad Tahir, Sheela Ramanna, Qian Liu

**Affiliations:** Department of Applied Computer Science, The University of Winnipeg, Winnipeg, MB R3B 2E9, Canada; Department of Applied Computer Science, The University of Winnipeg, Winnipeg, MB R3B 2E9, Canada; Department of Applied Computer Science, The University of Winnipeg, Winnipeg, MB R3B 2E9, Canada; Department of Biochemistry and Medical Genetics, University of Manitoba, Winnipeg, MB R3E 3N4, Canada

## Abstract

**Motivation:**

$N^{6}-\text{methyladenosine}$ ($m^{6}A$) is the most abundant internal modification in eukaryotic mRNA and plays essential roles in post-transcriptional gene regulation. While several deep learning approaches have been proposed to predict $m^{6}A$ sites, most suffer from limited chromosome-level generalizability due to evaluation on randomly split datasets.

**Results:**

In this study, we propose two novel hybrid deep learning models—Hybrid Model and Hybrid Deep Model—that integrate local sequence features (*k*-mers) and contextual embeddings via convolutional neural networks to improve predictive performance and generalization. We evaluate these models using both a Random-Split strategy and a more biologically realistic Leave-One-Chromosome-Out setting to ensure robustness across genomic regions. Our proposed models outperform the state-of-the-art m6A-TCPred model across all key evaluation metrics. Hybrid Deep Model achieves the highest accuracy under Random-Split, while Hybrid Model demonstrates superior generalization under Leave-One-Chromosome-Out, indicating that deep global representations may overfit in chromosome-independent settings. These findings underscore the importance of rigorous validation strategies and offer insights into designing robust $m^{6}A$ predictors.

**Availability and implementation:**

Source code and datasets are available at: https://github.com/malikmtahir/LOCO-m6A

## 1 Introduction

$N^{6}-\text{methyladenosine}$ ($m^{6}A$) is the most prevalent internal modification in eukaryotic messenger RNA (mRNA) and plays a pivotal role in regulating gene expression, RNA stability, splicing, and translation (Wang *et al.* 2022). As a dynamic and reversible epigenetic marker at the RNA level, $m^{6}A$ influences a wide range of post-transcriptional regulatory processes without altering the underlying nucleotide sequence (Zhao *et al.* 2017). Recent studies have demonstrated that dysregulation of $m^{6}A$ modifications is associated with various human diseases, including cancer, neurological disorders, and metabolic conditions (Jiang *et al.* 2021). For example, $m^{6}A$ RNA methylation plays a pivotal regulatory role in tumor immunity and is a promising target for cancer immunotherapy (Chen *et al.* 2025). As such, the accurate identification of $m^{6}A$ sites is crucial for advancing our understanding of gene regulation, its implications in health and disease, and for identifying potential therapeutic targets. Experimental approaches such as Mazter-Seq (Garcia-Campos *et al.* 2019), m6A-Seq (Dominissini *et al.* 2012), MeRIP sequencing (Meyer *et al.* 2012), miCLIP-m6A (Linder *et al.* 2015), and DART-Seq (Meyer 2019) have provided high-resolution maps of $m^{6}A$ modifications across different tissues and conditions. However, these techniques are labor-intensive, costly, and limited by resolution and reproducibility. Consequently, there is an increasing demand for computational methods that can reliably predict $m^{6}A$ sites from primary sequence data (Maestri *et al.* 2024).

Recent advances in machine learning and deep learning have shown promise in identifying complex patterns from biological sequences. For example, Chen *et al.* (2019) introduced a machine learning-based model, WHISTLE, to predict human $m^{6}A$ sites using various genomic features, achieving state-of-the-art performance with an Area Under the Receiver Operating Characteristic Curve (AUC-ROC) ranging from 0.880 to 0.948. Wang *et al.* (2023) proposed a higher order fuzzy inference system based on multiple deep kernel learning called DMKL-HFIS to predict DNA N4-methylcytosine (4mC) sites using fuzzy logic deep learning methods. The DMKL-HFIS approach merged a traditional feature extraction method, namely position-specific trinucleotide propensity, to preprocess and extract feature maps from benchmark datasets. By merging deep learning with fuzzy logic and traditional features, the method obtained higher prediction performance compared to existing models. Rehman *et al.* (2022) introduced a computational model called i6mA-Caps using a CapsuleNet-based framework to predict N6-methyladenine ($m^{6}A$) sites from DNA sequences. Huang *et al.* (2024) introduced a deep learning model based on a Convolutional Neural Network (CNN) and a bidirectional Gated Recurrent Unit (GRU). The model used several feature extraction methods, including one-hot encoding, dinucleotide one-hot encoding, nucleotide chemical properties, and sequence embeddings. Wang and Liu (2025), introduced a hybrid based deep learning model called m6A-SPP for the identification of m6A sites by integrating multi-source biological features. In sequence-based feature used a pretrained DNABERT model with CNN to process RNA sequence representations while in physicochemical-based feature computes embeddings by incorporating three key physicochemical properties of RNA such as Electron-Ion Interaction Potential (EIIP), Normalized Chemical Properties (NCP), and Dinucleotide Physicochemical Properties (DPP). Zhou *et al.* (2016), designed a random forest-based model called Sequence-based RNA Adenosine Methylation site Predictor (SRAMP) to identify mammalian $m^{6}A$ sites using sequence-derived features encoding schemes such as position nucleotide sequence patterns, spectrum features and K-Nearest Neighbors (KNN) information, and reported performance with AUC-ROC of 0.784 and 0.633 on mammalian and yeast datasets, respectively. Su *et al.* (2025), designed a computational framework called MST-m6A based on deep learning approach to predict m6A modification sites across diverse cellular contexts, containing multiple tissues and cell lines. The MST-m6A model reported performance with an accuracy range from 0.6098 to 0.8855 across all datasets. Zhang *et al.* (2021) introduced an ensemble deep learning model called EDLm6APred for the identification of $m^{6}A$ sites on messenger RNA using various feature encoding schemes such as one-hot encoding, RNA word embedding, and word2vec. The output of these encoding features was further processed by a BiLSTM model for prediction. The model obtained AUC-ROC of 0.8660, 0.8588, and 0.8605 on human, mouse, and mix datasets, respectively. Fan *et al.* (2023) presented ELMo4m6A model, which combined a CNN and the Long Short-Term Memory (LSTM) model to identify $m^{6}A$ sites. More recently, Tu *et al.* (2024) developed a framework called m6A-TCPred to predict human tissue-conserved $m^{6}A$ sites, based on traditional feature engineering methods—such as electron–ion interaction pseudopotential, chemical properties of nucleotides, and Pseudo K-tuple Nucleotide Composition—as well as machine learning-extracted features from genomic sequences. They explored various machine learning algorithms, including support vector machines, naïve Bayes, and generalized linear models, and reported state-of-the-art performance with AUC-ROCs of 0.871 and 0.879.

Despite the growing success of deep learning models in predicting $m^{6}A$ sites, a key limitation that remains largely unaddressed is the lack of chromosome-level generalizability. Most existing methods are trained and evaluated using random splits of the data, where training and testing samples often come from the same or similar genomic regions. This setup can lead to data leakage and overly optimistic performance estimates, as the model may learn region-specific biases rather than biologically meaningful patterns (Fine *et al.* 2019). In real-world applications, however, we often need to predict $m^{6}A$ sites in previously unseen chromosomes or genomic contexts, such as newly sequenced individuals, uncharacterized disease loci, or across species. Therefore, it is essential to evaluate models in a more stringent and realistic setting that simulates this scenario (Fine *et al.* 2019).

An important consideration in predicting $m^{6}A$ modifications is the relative importance of local versus global sequence features. $m^{6}A$ sites are strongly influenced by local sequence patterns and often appear in conserved motifs such as DRACH (D = A/G/U, R = A/G, H = A/C/U) (Su *et al.* 2025). The surrounding nucleotide context—typically within a 5–50 nucleotide window—provides critical information for identifying potential methylation sites (Zhang and Hamada 2018). However, global features also contribute meaningful insights, particularly for enhancing generalizability and capturing broader regulatory or structural context. Therefore, a well-balanced integration of both local and global features is essential to achieve high prediction accuracy while ensuring the model can generalize effectively across different genomic regions or conditions.

To address these limitations, we propose a novel hybrid representation learning framework for the prediction of human $m^{6}A$ modifications, with an emphasis on the balance of local and global features and chromosome-level generalizability. Our model integrates both local sequence features (k-mer) and proper scope of contextual embeddings (deep-CNN) to capture multi-scale patterns relevant to $m^{6}A$ modifications. Through comprehensive benchmarking and cross-chromosome validation, we demonstrate that our approach significantly outperforms existing methods and offers robust predictive power across diverse genomic landscapes. By focusing on chromosome-level generalizability, we aim to ensure that our model captures robust and transferable features, ultimately increasing its utility in diverse biological and clinical contexts.

## 2 Materials and methods

**Figure 1.**
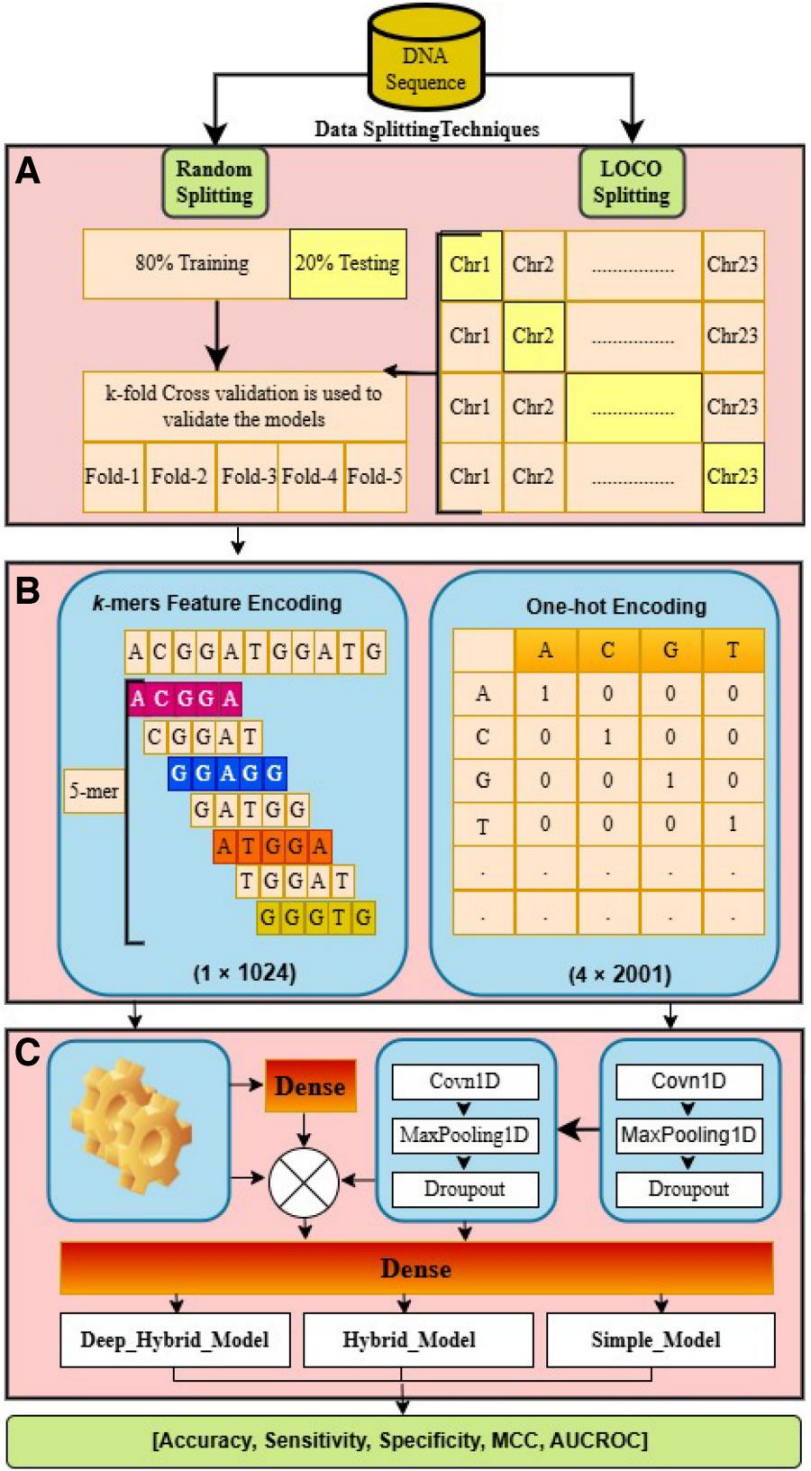
Overview of the proposed computational framework for $m^{6}A$ site prediction. (A) The process begins with a mRNA sequence input, which is divided using two data splitting strategies: Random-Split (RS) (80% training, 20% testing) with five-fold cross-validation, and Leave-One-Chromosome-Out (LOCO) validation, where one chromosome is used for testing and the remaining for training in each iteration. (B) Two types of feature encoding are applied to the genomic sequences: (1) *k*-mer encoding (with k=5), which captures the frequency of 5-length nucleotide subsequences and results in a 1×1024 feature vector; and (2) one-hot encoding, which represents each nucleotide (A, C, G, T) as a binary vector, forming a 4×2001 matrix for sequences of length 2001. (C) These encoded features are passed through different neural network configurations. The k-mer features are processed through dense layers, while one-hot encoded inputs are passed through stacked 1D convolutional layers (Conv1D), followed by max-pooling and dropout layers. The outputs from these pathways are concatenated and passed through a final dense layer for classification. Three model variants are considered: Deep-Hybrid-Model (DHM), Hybrid-Model (HM), and Simple-Model (SM). Model performance is evaluated using metrics including accuracy, sensitivity, specificity, MCC, and AUC-ROC.

An efficient and robust feature extraction and classification framework was proposed for $m^{6}A$ in this study. The framework was evaluated using both Random-Split (RS) and Leave-One-Chromosome-Out (LOCO) validation strategies. The overall workflow is illustrated in Fig. 1.

### 2.1 Data splitting

An $m^{6}A$ benchmark dataset (Tu *et al.* 2024) was used in this study. This dataset contains 268 115 base-resolution $m^{6}A$ sites (denoted as *D*), which were extracted from the $m^{6}A$-Atlas database (Tang *et al.* 2021). The dataset includes both positive and negative tissue-specific site sequences: the set of positive samples (*P*) consists of 10 424 sequences, while the set of negative samples (*N*) consists of 54 949 sequences. The full dataset *D* represents the union of both sets, i.e. D=P∪N, where ∪ denotes the union of sets.

Since the negative dataset (*N*) is approximately five times larger than the positive dataset (*P*), we randomly split *N* into five equal subsets: $N_{1},N_{2},N_{3},N_{4},N_{5}$. Each subset $N_{i}$ is of equal size:


(1) $$N_{i}=\frac{N}{5}$$

Each sub-dataset $N_{i}$ is then paired with set *P* to create five datasets ($D_{i}$) with 1:1 positive-to-negative ratio:


(2) $$D_{i}=P\cup N_{i}\;\text{for}\;\text{each}\;i=1,2,3,4,5$$

Since we used two evaluation strategies—RS and LOCO—they involved different data splitting procedures. For RS, each dataset $D_{i}$ was randomly split into 80% for training and 20% for independent testing. A five-fold cross-validation approach was then applied to the training set, where four-folds were used for training and one-fold for validation in each iteration. For LOCO, each $D_{i}$ was first divided into 23 subsets based on chromosome identifiers (chr1 to chr23) (Tahir *et al.* 2025). In each iteration, one chromosome was held out as the test set, while the remaining 22 chromosomes were used for training. Within the training set, five-fold cross-validation was applied to train and validate the model. This process was repeated for each chromosome, resulting in 23 independently trained models. The final performance was reported as the average across all 23 test chromosomes. An illustration of LOCO procedure is shown in Algorithm 1.

Algorithm 1. LOCO Training and Evaluation

1: Split the dataset into 23 subsets by chromosome: chr1, chr2, …, chr23.

2: **for** each chromosome $C_{i}$ in {chr1, …, chr23} **do**

3:   Use $C_{i}$ as the test set.

4:   Use the other 22 chromosomes as the training set.

5:   Train the model using five-fold cross-validation on the training set.

6:   Test the model on $C_{i}$.

7: **end for**

8: Compute the average performance over all 23 test sets.

### 2.2 Encoding scheme

Feature extraction plays a crucial role in identifying informative patterns from biological sequences. In this study, we employed two encoding methods: *k*-mer composition (Chen *et al.* 2014, Guo *et al.* 2014) and one-hot encoding combined with CNN (Tahir *et al.* 2024). The *k*-mer nucleotide composition is one of the most widely used sequence-based feature extraction techniques. It captures the frequency of all possible nucleotide subsequences of length *k* within an $m^{6}A$ sequence using a sliding window approach. Let the input sequence *S* be composed of four nucleotides from the set Σ={A,C,G,T}. For example, the $m^{6}A$ sequence CAAATGTACG (length = 10) can be represented by overlapping 5-mers: CAAAT, AAATG, AATGT, ATGTA, TGTAC, and GTACG, resulting in 10−5+1=6 5-mers. Across all possible combinations of nucleotides, there are $|\Sigma {|}^{k}=4^{5}=1024$ distinct 5-mers. However, any single DNA/RNA sequence typically contains only a subset of these, depending on its length and composition. In this study, the frequency (*f*) of each 5-mer was used as a numerical feature to represent the input sequence. Each $m^{6}A$ sequences are transformed into $4^{k}$ = $4^{5}$ = 1024-dimensional vector in Equation 3.


(3) $$k-\text{mer}\;\text{feature}\;\text{vector}=[f(K_{1}),f(K_{2}),\ldots ,f(K_{1024})]$$

One-hot encoding is a commonly used method to represent nucleotide sequences in a machine-readable format, where each nucleotide is mapped to a unique binary vector. For $m^{6}A$ sequences, this encoding converts the input sequence into a matrix with four channels, corresponding to the four nucleotides: *A*, *C*, *G*, and *U*. In this study, each $m^{6}A$ sequence is of fixed length L=2001. Each nucleotide in the sequence is encoded as a 4-dimensional binary vector: A as (1,0,0,0), C as (0,1,0,0), G as (0,0,1,0), and T as (0,0,0,1). As a result, each sequence is represented as a 4×L matrix, where the four rows correspond to the nucleotide channels and the columns represent the position in the sequence. This structured representation allows deep learning models, particularly CNNs, to effectively capture local sequence patterns and positional dependencies within the input. By adjusting the depth and architecture of the CNN and fully connected (dense) layers, we can control the size of the receptive field. This enables the model to learn both fine-grained (local) features and broader (global) contextual signals. Striking the right balance between local and global representation is essential for accurately modeling sequence characteristics, such as those associated with $m^{6}A$ modifications.

### 2.3 Validation: random split and leave one chromosome out

Figure 1 illustrates our proposed hybrid framework composed of the following layers: input layer, two 1D convolution layers, max-pooling layer, dropout layer, and fully connected layer for analyzing the sequence input. In the first layer of convolution, we define the number of filters as 64 with a kernel size of 13 and a stride of 1. Similarly, the second layer of convolution uses 128 filters with a kernel size of 23 and a stride of 1. These convolutional layers contain a rectified linear unit (ReLU) as activation function ReLU(y)=max(0,y), which converts negative values to zero. Then, a max-pooling layer with a window size of 15 is applied, which reduces the output dimension of the convolution layers to prevent overfitting. The next step is to concatenate the feature vectors of $m^{6}A$ sequences obtained from convolution layers with *k*-mer (5-mer) encoded features. These encodings are then input to the dropout layer and batch normalization, respectively. The dropout and batch normalization are regularization methods frequently used in neural networks to avoid overfitting. In this study, we set the drop probability to 0.5 in every dropout layer. The output of the batch normalization layer is input to the flatten layer, which converts feature maps into a single long feature vector. Finally, the produced feature vector is input to the dense layer, which is activated by the sigmoid activation function to predict $m^{6}A$ sites.

In this study, we trained six variants of models under two evaluation settings (RS and LOCO). These models fall into three categories based on their architecture: the Simple Model (RS-SM and LOCO-SM), the Hybrid Model (RS-HM and LOCO-HM), and the Deep Hybrid Model (RS-DHM and LOCO-DHM).

- Simple Model (SM): This model takes one-hot encoded $m^{6}A$ sequences as input. The encoded sequences are passed through a stack of 1D convolutional layers to automatically extract local features, followed by a dense layer with a sigmoid activation function for final classification.
- Hybrid Model (HM): In addition to one-hot encoded inputs processed via convolutional layers, this model incorporates handcrafted *k*-mer (5-mer) features. The learned CNN-based features and *k*-mer features are concatenated and fed into a dense layer for classification using a sigmoid activation function.
- Deep Hybrid Model (DHM): This model extends the HM by first passing the *k*-mer features through a fully connected layer with 64 neurons and ReLU activation, enabling higher-level abstraction of handcrafted features. These transformed features are then merged with the CNN-derived features and passed through a final dense layer with a sigmoid activation function for prediction.

Table 1 presents the layer-wise input and output dimensions for each of the three model types. In this study, all models were implemented in Python using the Keras library. The training was conducted using the Adam optimizer with a learning rate of 0.001, a batch size of 64, and a total of 70 training epochs. A dropout rate of 0.5 was applied for regularization, as summarized in Table 2. Since $m^{6}A$ site prediction is a binary classification task, all models were trained to minimize the binary cross-entropy loss function. The models were evaluated using RS and LOCO approaches described in Section Data splitting. In addition to these ablation analysis, the proposed hybrid models were also compared against the existing *m6A-TCPred* model (Tu *et al.* 2024).

**Table 1.** Details of proposed three hybrid models, such as simple model (RS-SM and LOCO-SM), hybrid model (RS-HM and LOCO-HM), and deep hybrid model (RS-DHM and LOCO-DHM).

Layers (type)	Output shape	Description
**Simple-Model (RS-SM and LOCO-SM)**		
Input 1	(None, 2001, 4)	Input of $m^{6}A$
Conv1D (64,23,1)	(None, 2001, 64)	Convolutional layer
MaxPooling1D (size = 15)	(None, 133, 64)	Max Pooling layer
Dropout (0.5)	(None, 133, 64)	Dropout layer
Conv1D (128,33,1)	(None, 133, 128)	Convolutional layer
MaxPooling1D (size = 15)	(None, 8, 128)	Max Pooling layer
Dropout (0.5)	(None, 8, 128)	Dropout layer
Flatten	(None, 1024)	Flatten layer
Dropout (0.5)	(None, 1024)	Dropout layer
Dense (1, sigmoid)	(None, 1)	Probability of m6A sites
**Hybrid-Model (RS-HM and LOCO-HM)**		
Input 1	(None, 2001, 4)	Input of $m^{6}A$
Input 2	(None, 1, 1024)	*k*-mer features of m6A
Conv1D (64,23,1)	(None, 2001, 64)	Convolutional layer
MaxPooling1D (size = 15)	(None, 133, 64)	Max Pooling layer
Dropout (0.5)	(None, 133, 64)	Dropout layer
Conv1D (128,33,1)	(None, 133, 128)	Convolutional layer
MaxPooling1D (size = 15)	(None, 8, 128)	Max Pooling layer
Dropout (0.5)	(None, 8, 128)	Dropout layer
Flatten	(None, 1024)	Flatten layer
Flatten	(None, 1024)	Flatten the *k*-mer features
Concatenate	(None, 2048)	Concatenate the features of *k*-mer and CNN
Dropout (0.5)	(None, 2048)	Dropout layer
Dense (1, sigmoid)	(None, 1)	Probability of $m^{6}A$ sites
**Deep Hybrid Model (RS-DHM and LOCO-DHM)**		
Input 1	(None, 2001, 4)	Input of $m^{6}A$
Input 2	(None, 1, 1024)	*k*-mer features of m6A
Conv1D (64,23,1)	(None, 2001, 64)	Convolutional layer
MaxPooling1D (size = 15)	(None, 133, 64)	Max Pooling layer
Dropout (0.5)	(None, 133, 64)	Dropout layer
Conv1D (128,33,1)	(None, 133, 128)	Convolutional layer
MaxPooling1D (size = 15)	(None, 8, 128)	Max Pooling layer
Dropout (0.5)	(None, 8, 128)	Dropout layer
Flatten	(None, 1024)	Flatten layer
Dense (64, relu)	(None, 1, 64)	Probability of $m^{6}A$ sites
Flatten	(None, 64)	Flatten the *k*-mer featrues
Concatenate	(None, 1088)	Concatenate the features of *k*-mer and CNN
Dropout (0.5)	(None, 1088)	Dropout layer
Dense (1, sigmoid)	(None, 1)	Probability of $m^{6}A$ sites

**Table 2.** Detailed summary of the applied parameters for training and testing the proposed model and experimental setup.

Category	Name of parameters	Configuration details
**Hardware**	CPU	Intel^®^ Xeon^®^ Silver 4214R, 12 cores @ 2.40 GHz
	GPU	NVIDIA GeForce RTX 3080, 64 GiB GDDR6X
	Storage	1 TB SSD
	RAM	256 GB DDR4
**Platform**	Operating System	Linux
	CUDA	12.1
**Software**	Python	3.9
	DL Framework	TensorFlow 2.17.0
	Loss function	Binary Crossentropy
	Optimizer	Adam (learning rate = 0.001)
	Batch Size	64
	No. of Epochs	70

### 2.4 Performance metrics

The predictive performance of the proposed models was measured using a comprehensive set of metrics, including accuracy, sensitivity (recall), specificity, Matthews correlation coefficient (MCC), and AUC-ROC. These metrics collectively assess different aspects of model performance, such as overall correctness (accuracy), the ability to detect true tissue conserved $m^{6}A$ sites (sensitivity), the ability to correctly identify non-tissue conserced $m^{6}A$ sites (specificity), the balance between true and false predictions (MCC), and the model’s discriminative ability across classification thresholds (AUC-ROC). To ensure statistical rigor, we also computed 95% confidence intervals for each metric, providing insights into the variability and reliability of the results. These intervals were estimated using repeated evaluation and bootstrap sampling techniques. A summary of all performance metrics (Chen *et al.* 2013, Kabir *et al.* 2018, Alam *et al.* 2020a, 2020b) along with their corresponding formulations and confidence interval estimates is presented in Table 3. This statistical analysis enhances the interpretability and robustness of the model evaluation.

**Table 3.** The details of the performance metrics.

Name	Formula	Description
Accuracy	$\frac{TN+TP}{TP+FN+TN+FP}$	TP: True Positive, TN: True Negative, FP: False Positive, FN: False Negative. The accuracy metric measures the ability of the model in making correct predictions
Sensitivity	$\frac{TP}{TP+FN}$	The sensitivity metric measures the ability of the model to predict positive instances
Specificity	$\frac{TN}{FP+TN}$	The specificity metric measures ability of the model to predict negative instances
MCC	$\frac{TP\times TN-FP\times FN}{\sqrt{\left(TN+FP)(TN+FN)(TP+FP)(TP+FN\right)}}$	The MCC metric provides a balanced evaluation, considering misclassifications in both classes
AUC-ROC	$\int_{0}^{1}SN(SP^{-1}d(x))dx$	The AUC-ROC metric plots the true positive rate against false positive rate
Confidence Interval	$\mu \;\pm \;(z\times \frac{\sigma }{\sqrt{S_{\mathrm{size}}}})$	where $S_{\mathrm{size}}$ is the total sample size of the observations, μ is the mean of the data, *z* is the critical value, and σ is the standard deviation of the data

## 3 Results and discussion

### 3.1 Feature visualization

**Figure 2.**
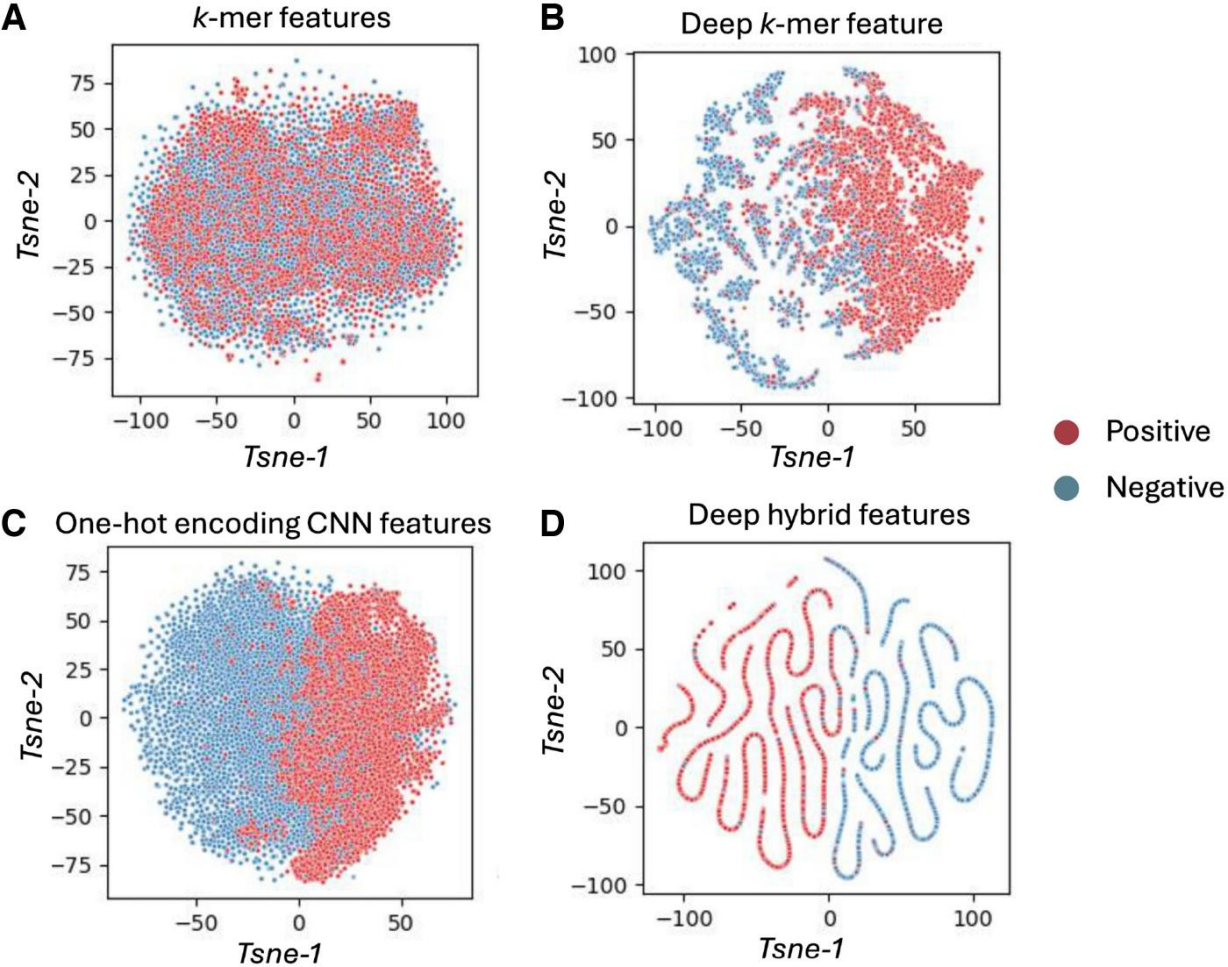
*t*-SNE visualizations of learned feature representations under different encoding schemes and model configurations. (A) Raw *k*-mer features without deep learning show minimal class separation. (B) Deep *k*-mer features, extracted using a fully connected layer, exhibit improved separability between positive and negative samples. (C) One-hot encoded features processed through CNN layers show moderate class separation. (D) Deep hybrid features, combining transformed *k*-mer and CNN-derived features, demonstrate the most distinct clustering of positive and negative samples, indicating the effectiveness of the proposed HD-$m^{6}A$ model in learning highly discriminative representations.

The learned features under different encoding schemes and model depths were visualized using t-distributed stochastic neighbor embedding (t-SNE) (Van der Maaten and Hinton 2008) and are shown in Fig. 2. t-SNE is a non-linear dimensionality reduction technique that preserves the local structure of high-dimensional data in a low-dimensional space, making it well-suited for evaluating the separability of features learned by deep models. As illustrated in Fig. 2, distinct patterns emerge across different feature extraction strategies. The raw *k*-mer features (Fig. 2A) show poor separation between positive and negative samples, indicating that this representation alone lacks discriminative power. In contrast, deep *k*-mer features obtained through a dense transformation layer (Fig. 2B) exhibit improved clustering, suggesting that the learned non-linear representations better capture class-specific signals. The one-hot encoded features processed through CNN layers (Fig. 2C) demonstrate more pronounced separation between the two classes, highlighting the effectiveness of convolutional layers in capturing local sequence patterns. Most notably, the deep hybrid features (Fig. 2D), which integrate both deep *k*-mer and CNN-based features, produce the clearest and most distinct clusters. This visualization confirms that the hybrid models effectively learn complementary information from both handcrafted and learned representations, resulting in highly discriminative feature embeddings that support superior classification performance.

### 3.2 Random split validation

Table 4 presents the performance of three model architectures, RS-SM, RS-HM, and RS-DHM, under the RS validation approach. In the training sets, the RS-DHM model demonstrated the highest performance across several key metrics, including accuracy (0.8863 ± 0.0023), MCC (0.7730 ± 0.0046), and AUC-ROC (0.9452 ± 0.0015), indicating robust overall learning. Interestingly, the RS-SM model achieved the highest sensitivity (0.9010 ± 0.0032), suggesting improved detection of positive $m^{6}A$ sites, though at the cost of lower specificity (0.8186 ± 0.0065). In contrast, the RS-HM model exhibited the highest specificity (0.8806 ± 0.0042), indicating better discrimination of negative cases but a slight trade-off in balanced classification performance compared to RS-SM. When evaluated on the independent test dataset, the RS-DHM model consistently outperformed the other models across most evaluation metrics, achieving the highest accuracy (0.8905 ± 0.0042), sensitivity (0.9046 ± 0.0044), MCC (0.7816 ± 0.0083), and AUC-ROC (0.9472 ± 0.0027).

**Table 4.** Performance of the models on the training and independent test datasets using the RS approach.

Datasets	Models	Accuracy	Sensitivity	Specificity	MCC	AUC-ROC
Training	RS-SM	0.8587 ± 0.0028	**0.9010 ± 0.0032**	0.8186 ± 0.0065	0.7210 ± 0.0053	0.9330 ± 0.0012
	RS-HM	0.8665 ± 0.0022	0.8519 ± 0.0075	**0.8806 ± 0.0042**	0.7331 ± 0.0044	0.9331 ± 0.0015
	RS-DHM	**0.8863 ± 0.0023**	0.8978 ± 0.0037	0.8755 ± 0.0027	**0.7730 ± 0.0046**	**0.9452 ± 0.0015**
Independent test	RS-SM	0.8590 ± 0.0028	0.9035 ± 0.0027	0.8167 ± 0.0079	0.7219 ± 0.0048	0.9350 ± 0.0016
	RS-HM	0.8709 ± 0.0046	0.8610 ± 0.0049	**0.8804 ± 0.0077**	0.7419 ± 0.0091	0.9363 ± 0.0029
	RS-DHM	**0.8905 ± 0.0042**	**0.9046 ± 0.0044**	0.8771 ± 0.0069	**0.7816 ± 0.0083**	**0.9472 ± 0.0027**

The best results for each metric are highlighted in bold.

These results highlight the superior generalization capability of the RS-DHM architecture. Overall, RS-DHM demonstrates the most balanced and reliable performance on both the training and test datasets, particularly in terms of classification robustness and generalizability. These findings underscore the impact of architectural design on predictive performance, with the deep hybrid structure of RS-DHM proving to be the most effective among the three. To further illustrate and compare the models’ classification capabilities, the AUC-ROC for RS-SM, RS-HM, and RS-DHM on both the training and test datasets are presented in the Supplementary Figure, available as supplementary data at *Bioinformatics Advances* online.

### 3.3 Leave one chromosome out validation

Table 5 presents the performance metrics of the models, LOCO-SM, LOCO-HM, and LOCO-DHM, evaluated using the LOCO approach on the benchmark dataset. For each model, 23 separate training and testing iterations were conducted, with one chromosome held out in each iteration. The final results represent the average performance across all chromosomes. On the training set, the LOCO-DHM model achieved the highest overall performance, with an accuracy of 0.9030 ± 0.0006, sensitivity of 0.9155 ± 0.0007, MCC of 0.8065 ± 0.0013, and AUC-ROC of 0.9563 ± 0.0004, indicating strong learning and internal generalization. While LOCO-HM achieved slightly lower overall metrics, it demonstrated relatively higher specificity, and LOCO-SM showed similar but slightly lower performance across all evaluation parameters. However, in the independent test dataset, LOCO-HM outperformed both LOCO-SM and LOCO-DHM in terms of accuracy, specificity, MCC, and AUC-ROC, suggesting that it generalizes better in the more realistic LOCO evaluation setting. Supplementary Figure, available as supplementary data at *Bioinformatics Advances* online shows the AUC-ROC curves for all models under the LOCO evaluation protocol.

**Table 5.** Performance of the models on the training and independent test datasets using the LOCO splitting approach.

Datasets	Models	Accuracy	Sensitivity	Specificity	MCC	AUC-ROC
Training	LOCO-SM	0.8768 ± 0.0006	0.9056 ± 0.0009	0.8496 ± 0.0016	0.7556 ± 0.0010	0.9443 ± 0.0003
	LOCO-HM	0.8771 ± 0.0004	0.8566 ± 0.0018	**0.8965 ± 0.0017**	0.7546 ± 0.0008	0.9429 ± 0.0002
	LOCO-DHM	**0.9030 ± 0.0006**	**0.9155 ± 0.0007**	0.8912 ± 0.0010	**0.8065 ± 0.0013**	**0.9563 ± 0.0004**
Independent test	LOCO-SM	0.8129 ± 0.0047	**0.7136 ± 0.0195**	0.8308 ± 0.0060	0.4496 ± 0.0204	0.8543 ± 0.0086
	LOCO-HM	**0.8430 ± 0.0050**	0.6394 ± 0.0237	**0.8795 ± 0.0053**	**0.4657 ± 0.0256**	**0.8611 ± 0.0086**
	LOCO-DHM	0.8214 ± 0.0066	0.5646 ± 0.0271	0.8672 ± 0.0060	0.3882 ± 0.0248	0.7913 ± 0.0137

The best results for each metric are highlighted in bold.

Overall, while DHM exhibits the best performance on the training set—with high accuracy, sensitivity, MCC, and AUC-ROC, as well as a balanced trade-off between sensitivity and specificity—it did not achieve the same level of success on the independent test dataset. Notably, DHM performs best under the RS validation strategy on both training and test sets. However, its performance drops substantially under the more rigorous and biologically realistic LOCO setting. This discrepancy raises important questions about the generalization capabilities of existing deep learning models for $m^{6}A$ prediction and emphasizes the need for more robust evaluation protocols in future studies.

**Figure 3.**
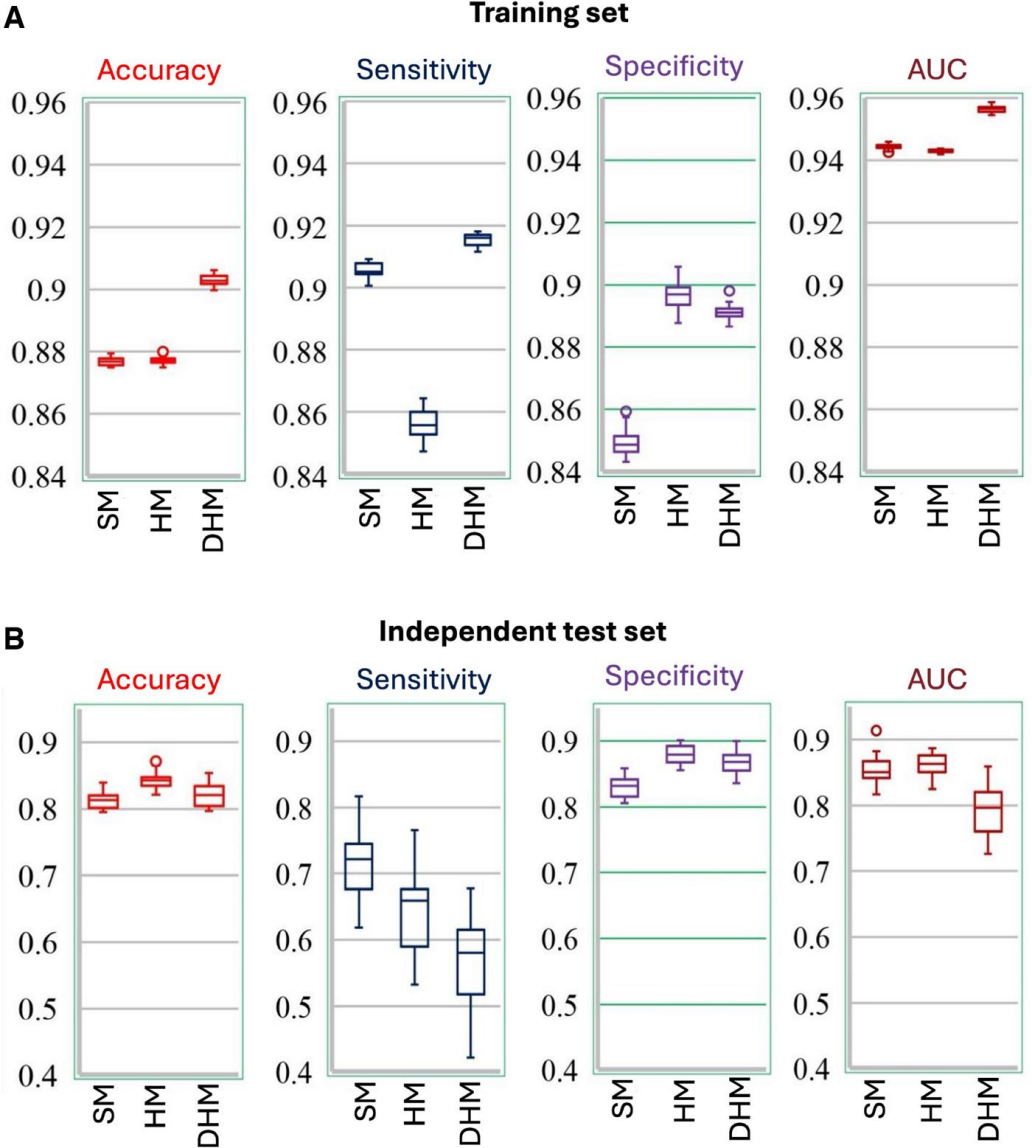
Box plots illustrating the variation in performance metrics—accuracy, sensitivity, specificity, and AUC-ROC—across the 23 chromosomes for each of the three kinds of features (SM, HM, and DHM), evaluated using the LOCO approach. (A) Performance distribution on the training set. DHM exhibits the highest and most consistent performance across all metrics, including accuracy, sensitivity, specificity, and AUC-ROC. (B) Performance distribution on the independent test set. While the DHM model maintains strong performance in some metrics, the HM model demonstrates better generalization in terms of accuracy, specificity, and AUC-ROC. These results highlight the differences in generalization behavior and metric trade-offs between simple, hybrid, and deep hybrid models under the LOCO setting.

To further assess model performance and variability across genomic regions, we examined the distribution of four key evaluation metrics—accuracy, sensitivity, specificity, and AUC-ROC—across all 23 chromosomes under the LOCO splitting scheme, as shown in Fig. 3. Figure 3A presents results on the training set, where the DHM model consistently outperformed SM and HM across most metrics, showing higher median values and tighter distributions, particularly for accuracy, sensitivity, and AUC-ROC. This suggests that DHM learns more robust and generalizable features during training. Figure 3B shows the performance on the independent test set, where a shift in relative performance is observed. While DHM continues to show competitive specificity, its performance varies more across chromosomes, especially in AUC-ROC and sensitivity. In contrast, the HM model achieves the highest median performance in accuracy, specificity, and AUC-ROC on the test set, indicating superior generalization under the LOCO setting. The SM model lags behind both HM and DHM across most metrics, except sensitivity. These findings highlight the trade-offs between model complexity and generalization, and reinforce the importance of chromosome-level evaluation in $m^{6}A$ site prediction.

### 3.4 Comparison with the benchmark model

Table 6 presents a comparative performance analysis between the benchmark model, m6A-TCPred (Tu *et al.* 2024), and our proposed models—HM and HDM—across all evaluation metrics under both RS and LOCO validation strategies. Under the RS approach, the HDM model achieved an accuracy of 0.886 on the training set, significantly outperforming m6A-TCPred, which obtained 0.792—a relative improvement of 9.4%. In addition, the proposed model achieved gains of 10.3%, 8.7%, and 18.9% in sensitivity, specificity, and MCC, respectively. The AUC-ROC, a key indicator of overall classification capability, also showed notable improvement: HDM achieved an AUC-ROC of 0.946, compared to 0.881 from m6A-TCPred, representing a 7.42% gain.

**Table 6.** Performance comparison between the benchmark model and the proposed models under RS and LOCO validation approaches on both the training and independent test datasets.

Models	Validation	Datasets	Accuracy	Sensitivity	Specificity	MCC	AUC-ROC
m6A-TCPred (Tu *et al.* 2024)	RS	Training	0.792	0.795	0.789	0.584	0.871
DHM-$m^{6}A$	RS		0.886	0.898	0.876	0.773	0.945
	LOCO		**0.903**	**0.916**	**0.891**	**0.807**	**0.956**
m6A-TCPred (Tu *et al.* 2024)	RS	Independent test	0.801	0.806	0.796	0.603	0.879
HM-$m^{6}A$	RS		**0.891**	**0.905**	**0.877**	**0.782**	**0.947**
	LOCO		0.843	0.640	0.867	0.466	0.861

The best performances for each metric are highlighted in bold.

On the more challenging LOCO evaluation, our proposed HM model maintained strong generalization with an accuracy of 0.903, outperforming m6A-TCPred by 11.1%. The performance gains were consistent across other metrics as well, with improvements of 12.1% in sensitivity, 10.2% in specificity, and 22.3% in MCC. AUC-ROC again reflected this superiority, with HM achieving 0.956, an 8.5% increase over the baseline model. On the independent test set under RS, our models outperformed m6A-TCPred by 9.0% in accuracy, 9.9% in sensitivity, 8.1% in specificity, 17.9% in MCC, and 6.8% in AUC-ROC. These consistent double-digit improvements across training and testing datasets confirm the effectiveness of the proposed architectures under RS.

However, it is important to note a key limitation of the RS approach: since the same chromosome may appear in both training and testing datasets, this setup introduces information leakage, which can lead to overly optimistic performance estimates. In contrast, the LOCO approach offers a more rigorous and biologically realistic evaluation by completely isolating chromosomes between training and testing. When applying LOCO to m6A-TCPred, we observed substantial performance degradation, with reductions of 7% in accuracy, 34% in sensitivity, 1% in specificity, 39.4% in MCC, and 5.57% in AUC-ROC. This drastic decline underscores the importance of using chromosome-level validation for a fair assessment of generalization capability in $m^{6}A$ site prediction models.

Similarly, on LOCO splitting approach the proposed models obtained 2% and 7.1% higher accuracy and specificity than benchmark m6A-TCPred model but lower sensitivity, MCC and AUC-ROC. While LOCO approach outcomes demonstrate slightly reduced performance, the proposed model still maintains a competitive performance, highlighting its robustness and generalization capability. These improvements confirm that HM-$m^{6}A$ and DHM-$m^{6}A$ are a significantly enhanced predictive model for $m^{6}A$ sites, making them more reliable tools for $m^{6}A$ predictions.

## 4 Conclusion

In this study, we introduced two novel hybrid deep learning models—HM and HDM—for the accurate prediction of tissue conserved $m^{6}A$ mRNA methylation sites. These models combine traditional *k*-mer features with one-hot encoded sequences processed through convolutional layers, enabling the integration of both handcrafted and automatically learned sequence features. To ensure rigorous performance evaluation, we employed two validation strategies: RS and LOCO. While RS is widely used, it may lead to inflated performance due to overlapping genomic regions between training and testing sets. LOCO, on the other hand, provides a more biologically realistic assessment by eliminating any chromosome-level data leakage.

Our results demonstrate that the HDM model achieves superior performance in the RS setting, leveraging its deep architecture to extract complex sequence patterns. However, under the stricter LOCO protocol, the HM model consistently outperforms HDM, suggesting that deeper, more global representations may be prone to overfitting in the absence of genomic overlap. This contrast highlights a critical insight: model complexity must be carefully balanced with generalization capability, especially in genomic applications where data dependencies are non-trivial.

The significance of this study lies in three key contributions. First, we present robust hybrid architectures that outperform a state-of-the-art benchmark (m6A-TCPred) across nearly all evaluation metrics. Second, we advocate for the adoption of LOCO as a standard evaluation strategy for genomic prediction tasks to avoid misleading conclusions about model performance. Third, by dissecting the behavior of models under both RS and LOCO, we provide a nuanced understanding of how feature encoding and model depth impact generalizability.

Overall, our work not only advances the state of the art in $m^{6}A$ site prediction but also provides critical guidance for building and evaluating deep learning models in bioinformatics. The proposed HM and HDM frameworks offer promising tools for downstream functional analysis and have the potential to support broader efforts in epitranscriptomic research and RNA-based therapeutics.lusions here.

## Supplementary Material

vbaf170_Supplementary_Data
