# Supplementary material for: Hybrid representation learning for human m6A modifications with chromosome-level generalizability
Source: Bioinform Adv. 2025 Jul 14;5(1):vbaf170. doi: 10.1093/bioadv/vbaf170 (PMC12288952; doi:10.1093/bioadv/vbaf170)
Supplement: vbaf170_Supplementary_Data [file vbaf170_supplementary_data.docx]

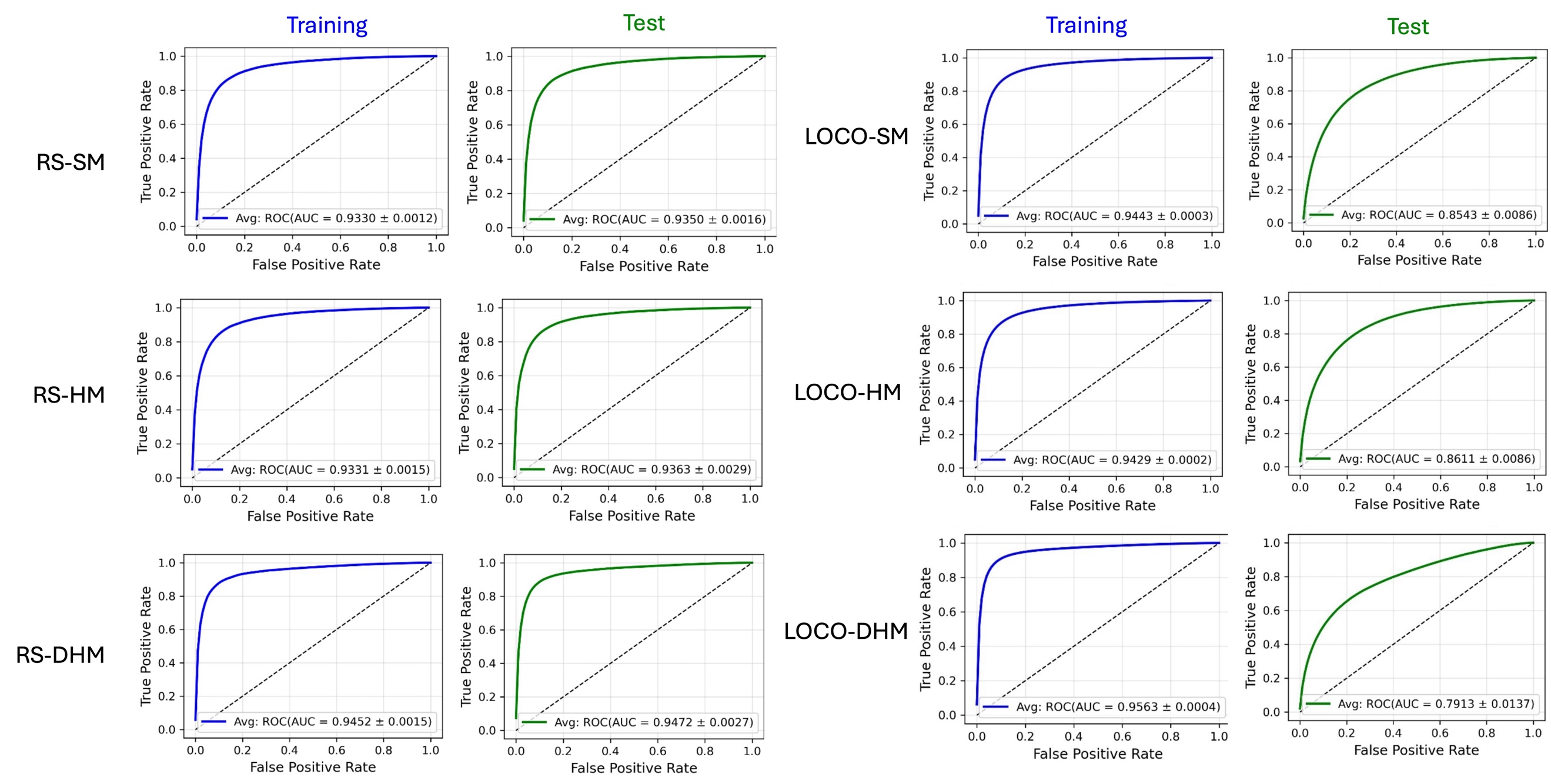


**Supplementary Figure.** Receiver Operating Characteristic (ROC) curves comparing the classification performance of three model architectures—Simple Model (SM), Hybrid Model (HM), and Deep Hybrid Model (DHM)—under two evaluation strategies: Random-Split (RS) and Leave-One-Chromosome-Out (LOCO). The left side of the figure shows results from the RS strategy, and the right side shows results from the LOCO strategy. Each row corresponds to one model, with training performance shown in blue and test performance in green. Average AUC values with standard deviations are displayed on each plot. The results demonstrate that while the DHM model performs best under the RS setting, the HM model shows superior generalization in the more realistic LOCO setting.
